# Supplementary material for: A time-resolved, multi-symbol molecular recorder via sequential genome editing
Source: Nature. 2022 Jul 6;608(7921):98–107. doi: 10.1038/s41586-022-04922-8 (PMC9352581; doi:10.1038/s41586-022-04922-8)

nature portfolio

https://doi.org/10.1038/s41586-022-04922-8

Supplementary information

A time-resolved, multi-symbol molecular recorder via sequential genome editing

In the format provided by the authors and unedited

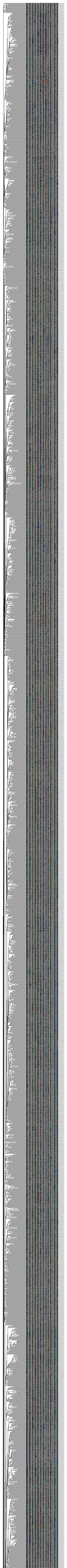

Supplement: Supplementary file 1 — Monophyletic cell lineage tree for 3,257 cells. [file 41586_2022_4922_MOESM1_ESM.pdf]
